# Exclusive Association of *p53* Mutation with Super-High Methylation of Tumor Suppressor Genes in the *p53* Pathway in a Unique Gastric Cancer Phenotype

**DOI:** 10.1371/journal.pone.0139902

**Published:** 2015-10-08

**Authors:** Mina Waraya, Keishi Yamashita, Akira Ema, Natsuya Katada, Shiro Kikuchi, Masahiko Watanabe

**Affiliations:** Department of Surgery, Kitasato University School of Medicine, Kitasato 1-15-1, Minami-ku, Sagamihara, Kanagawa 252-0374, Japan; The Chinese University of Hong Kong, HONG KONG

## Abstract

**Background:**

A comprehensive search for DNA methylated genes identified candidate tumor suppressor genes that have been proven to be involved in the apoptotic process of the *p53* pathway. In this study, we investigated *p53* mutation in relation to such epigenetic alteration in primary gastric cancer.

**Methods:**

The methylation profiles of the 3 genes: *PGP9*.*5*, *NMDAR2B*, and *CCNA1*, which are involved in the p53 tumor suppressor pathway in combination with *p53* mutation were examined in 163 primary gastric cancers. The effect of epigenetic reversion in combination with chemotherapeutic drugs on apoptosis was also assessed according to the tumor *p53* mutation status.

**Results:**

*p53* gene mutations were found in 44 primary gastric tumors (27%), and super-high methylation of any of the 3 genes was only found in cases with wild type *p53*. Higher *p53* pathway aberration was found in cases with male gender (p = 0.003), intestinal type (p = 0.005), and non-infiltrating type (p = 0.001). The *p53* pathway aberration group exhibited less recurrence in lymph nodes, distant organs, and peritoneum than the *p53* non-aberration group. In the NUGC4 gastric cancer cell line (*p53* wild type), epigenetic treatment augmented apoptosis by chemotherapeutic drugs, partially through *p53* transcription activity. On the other hand, in the KATO III cancer cell line (*p53* mutant), epigenetic treatment alone induced robust apoptosis, with no trans-activation of *p53*.

**Conclusion:**

In gastric cancer, *p53* relevant and non-relevant pathways exist, and tumors with either pathway type exhibited unique clinical features. Epigenetic treatments can induce apoptosis partially through *p53* activation, however their apoptotic effects may be explained largely by mechanism other than through *p53* pathways.

## Introduction

DNA methylation plays a central role in gene silencing of tumor suppressor genes in human cancer. A cancer-specific methylation gene is a rare entity, and frequent aberration of methylation in primary tumor tissues is even more rare [1, 2]. We identified cancer-specific methylated genes in each organ using a pharmacological unmasking microarray (PUM) [1, 2] and a modified PUM [3–5]. We identified many candidate tumor suppressor genes (TSGs), such as *PGP9*.*5* in head and neck squamous cell carcinoma (HNSCC) [2], esophageal SCC (ESCC) [6], gastric cancer [4], and other cancers [7], *NMDAR2B* in ESCC [3] and gastric cancer [8], and *CCNA1* in HNSCC [2]. The methylation profiles of these genes have been validated by other groups and/or even in other cancers [9–14]. Genes that showed over 60% methylation in tumor tissues were designated as highly relevant methylated genes (HRMGs) [15]. Moreover, we further compared the frequency of such aberrant methylation of candidate HRMGs with other reports of gastric cancer [16], and gene candidates were narrowed down to specific genes.

**Fig 1:**
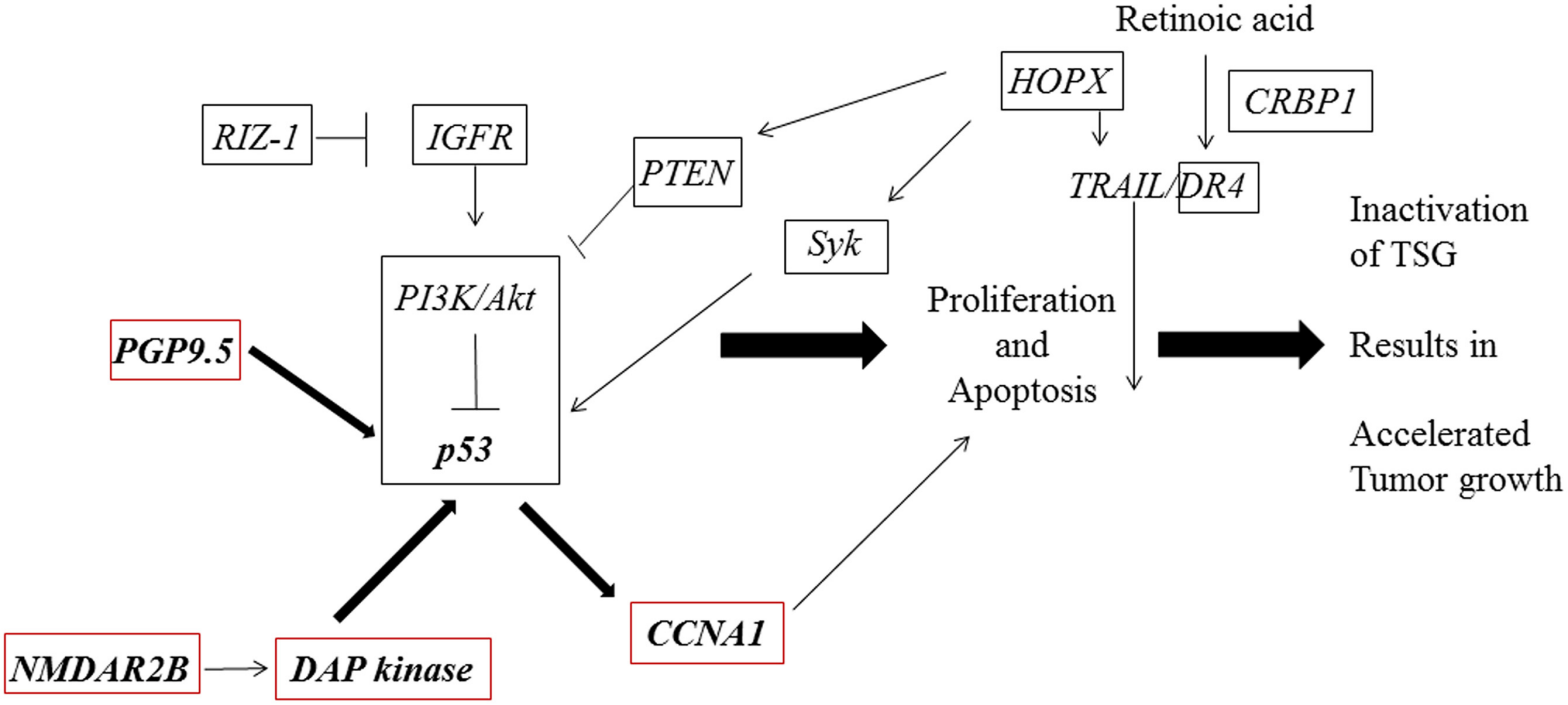
Epigenetic conversion in the *p53* pathway.

Most importantly, these candidate tumor suppressor genes had been reported to be in the *p53* tumor suppressor pathway (Fig 1). For example, *PGP9*.*5* directly interacts with *p53* and stabilizes *p53* by inhibiting its degradation through the ubiquitination pathway in hepatocellular [17], breast [18], and nasopharyngeal cancer [19]. *NMDAR2B* induces apoptosis through its direct interaction with *DAPK* [20], which, in turn, has been demonstrated to counteract oncogene-induced transformation by activating a *p19ARF*/*p53*-dependent apoptotic checkpoint [21]. *CCNA1* is a *p53*-induced gene that mediates apoptosis, G2/M arrest, and mitotic catastrophe in human renal, ovarian, and lung cancer cells [22]. Hence, the *p53* pathway is ablated in tumor tissues of primary cancers in an epigenetic manner together with wild type *p53*, however there has been no report regarding the association of *p53* mutation and epigenetic alterations in primary tumor tissues.

In this study, we investigated the DNA methylation status of genes in the *p53* pathway that are abnormally regulated in an epigenetic manner in primary gastric cancer, and compared their methylation pattern with the *p53* mutation status in order to determine the clinical significance of *p53* aberration phenotypes.

## Methods

### Cell lines and tissue samples

The gastric cancer cell lines, KatoIII, NUGC4, AZ521, and SH10 were purchased from the RIKEN BioResource Center (Ibaraki, Japan). And the hepatocellular carcinoma cell line HepG2 was purchased from American Type Culture Collection (Manassas, VA). These cell lines except AZ521 and HepG2 were grown in RPMI 1640 medium (GIBCO, Carlsbad, CA) supplemented with 10% fetal bovine serum. AZ521 and HepG2 were grown in DMEM medium (GIBCO), supplemented with 10% FBS.

Pairs (n = 163) of formalin-fixed, paraffin-embedded (FFPE) tumor tissue and corresponding normal mucosal specimens obtained at least 5 cm from the tumor edge were obtained from patients undergoing surgery between January 1, 2000, and December 31, 2010. All of the patients with stage II/III GC had undergone a potentially curative resection for the primary GC, and underwent adjuvant S-1 chemotherapy after surgery (S-1 standard treatment). Neo-adjuvant therapy was not performed in this patient cohort. Tumors were classified using the TNM classification according to the 7^th^ edition of the Union for International Cancer Control (UICC) and the 14^th^ edition of the Japanese Classification of Gastric Carcinoma (JCGC). The patients’ characteristics are depicted in Table 1. All tissue samples were collected at the Kitasato University Hospital, and written informed consent was obtained from all patients and healthy donors before sample collection. The present study was approved by the Ethics Committee of Kitasato University.

**Table 1:** Distribution of clinical and pathological factors for correlation with gene & methylation status and univariable prognostic analysis in 163 pStageII/III gastric cancer with gastrectomy and subsequent S-1 treatment.

variable	*p53* mutant	*p53* WT/ SHM*	*p53* WT w/o SHM**	P value^#^	RFS***	P value	OS****	P value
**Gender**				**0.0003**		**0.03**		**0.1**
** Male**	**40**	**25**	**48**		**60.7**		**57.8**	
** Female**	**4**	**9**	**37**		**86.7**		**81.9**	
**Age**				**0.08**		**0.009**		**0.001**
** <67**	**19**	**20**	**54**		**80.0**		**78.5**	
** ** **≥** **67**	**25**	**14**	**31**		**45.4**		**42.9**	
**Tumor location**				**0.8**		**0.06**		**0.1**
** Upper**	**12**	**11**	**29**		**52.1**		**58.7**	
** Middle**	**22**	**14**	**35**		**77.6**		**68.7**	
** Lower**	**10**	**9**	**21**		**85.3**		**76.6**	
**Lauren's histology**				**0.005**		**0.1**		**0.3**
** Diffuse type**	**22**	**19**	**65**		**77.9**		**67.7**	
** Intestinal type**	**22**	**15**	**20**		**52.1**		**75.4**	
**pT factor (14th JGCA/7th UICC)**				**0.3**		**0.08**		**0.4**
** T2**	**9**	**5**	**14**		**92.3**		**87.6**	
** T3**	**12**	**5**	**16**		**85.4**		**77.2**	
** T4a**	**23**	**23**	**55**		**62.3**		**62.1**	
** T4b**	**0**	**1**	**0**		**50.0**		**50.0**	
**pN factor (14th JGCA/7th UICC)**				**0.2**		**0.001**		**0.06**
** N0**	**2**	**4**	**16**		**66.7**		**100**	
** N1**	**9**	**11**	**23**		**91.4**		**84.6**	
** N2**	**14**	**7**	**17**		**76.5**		**63.2**	
** N3**	**19**	**12**	**29**		**57.0**		**52.8**	
**pStage (14th JGCA/7th UICC)**				**0.6**		**<0.0001**		**0.03**
** IIA**	**4**	**2**	**7**		**100**		**100**	
** IIB**	**5**	**6**	**24**		**76.8**		**88.6**	
** IIIA**	**13**	**11**	**19**		**87.8**		**75.8**	
** IIIB**	**11**	**7**	**17**		**71.8**		**66.7**	
** IIIC**	**11**	**8**	**18**		**45.6**		**43.4**	
**Infiltration pattern**				**0.001**		**0.9**		**0.7**
** α**	**2**	**5**	**4**		**69.3**		**87.5**	
** β**	**24**	**20**	**27**		**66.9**		**76.5**	
** γ**	**18**	**9**	**54**		**72.6**		**62.7**	
**Lymphatic permeation**				**0.1**		**0.1**		**0.2**
** No**	**2**	**0**	**7**		**100**		**100**	
** Yes**	**42**	**34**	**78**		**69.6**		**67.3**	
**Vascular permieation**				**0.2**		**0.1**		**0.03**
** No**	**2**	**2**	**11**		**93.3**		**100**	
** Yes**	**42**	**32**	**74**		**66.3**		**61.5**	

*p53 wild type with super-high methylation;

**p53 wild type without super-high methylation;

***relapse free survival;

****overall survival;

^#^ vs. *p53* mutat, Mann-Whtney U test.

### Analysis of mutated *p53* genes using single-strand conformation polymorphism (SSCP)

Mutations in exons 5, 6, 7 and 8 of the *p53* gene were screened by non-radioactive single-strand conformation polymorphism (SSCP) analysis, which was performed using our previously established methods [23]. PCR product samples of 10 μl were diluted threefold with gel-loading buffer (95% deionized formamide, 20 mmol/L EDTA, 0.01% bromophenol blue, and 0.01% xylene cyanol) and heated to 95°C for 10 min, followed by quenching on ice. Aliquots of 3 μl were applied to modified polyacrylamide gels (PAFG: 18% polyacrylamide-bis (49:1), 0.5x TBE, 10% glycerol, 10% formamide, 0.05% ammonium persulfate, and 30 ml TEMED) of dimensions 120 mm x 150 mm x 0.35 mm. Electrophoresis was performed with 1.5x TBE running buffer at 500 V and 30 mA for 1 hour at room temperature. Bands were detected by staining gels using a silver stain plus kit (Bio-Rad, Hercules, CA), followed by fixation, rinsing, development, and stopping of the reaction. Mutated bands detected with PCR-SSCP were evident at 1:64 dilution of mutated alleles.

### Bisulfite treatment of extracted DNA

Genomic DNA from FFPE tissues and cell lines was extracted using the QIAamp DNA FFPE Tissue kit (QIAGEN Sciences, Maryland, MD) and the QIAamp DNA Mini Kit (QIAGEN) according to the manufacturer’s protocols. For DNA denaturing, 2 μg of genomic DNA was incubated with 5 μg of salmon sperm DNA in 0.3 mol/l NaOH for 20 minutes at 50°C. The DNA sample was then diluted with 500 μl of a solution containing 2.5 mol/l sodium metabisulfite (Sigma-Aldrich Inc., St. Louis, MO)/ 125 mmol/l hydroquinone (Sigma)/ 0.4 mol/l sodium hydroxide solution, and was incubated at 70°C for 1.5 hours. The sample was then applied to a column (Wizard DNA Clean-UP System, Promega Inc., Madison, WI), incubated with 0.3 mol/l NaOH for 10 minutes, and subsequently treated with 3 mol/l ammonium acetate for 5 minutes. The sample was precipitated in 100% ethanol, and the DNA was resuspended in 50 μl LoTE containing 10 μM Tris-HCl, pH 8 and 2.5 μM ethylene diamine tetra acetic acid (EDTA), pH 8, and was subsequently amplified by a polymerase chain reaction (PCR). Bisulfite treatment results in the chemical modification of unmethylated, but not methylated, cytosines to uracils, allowing the distinction between methylated and unmethylated genomic DNA.

### Quantitative-methylation-specific PCR (Q-MSP)

For quantitative methylation analysis, TaqMan methylation specific PCR (Q-MSP) was carried out using the iQ™ Multiplex Powermix (Bio-Rad) in triplicate on the iCycler iQ™ Real-Time PCR Detection system (Bio-Rad). PCR conditions and sequences are provided in S1 Table. Serial dilutions of bisulfite modified CpGenome universal methylated DNA (Chemicon International, Temecula, CA) were used to construct the calibration curve on each plate as methylation positive controls and CpGenome universal unmethylated DNA (Chemicon International) was used as the negative control. The methylation value (TaqMeth value) was defined as the ratio of methylated *PGP9*.*5*, *NMDAR2B*, *CCNA1*, or *DAPK* normalized to methylated *β-actin*, which was then multiplied by 100.

### Immunohistochemical staining of PGP9.5, NMDAR2B, and CCNA1

For immunostaining, antigen unmasking was performed with autoclave soaking, endogenous peroxidase activity was blocked by incubation in 3% H_2_O_2_/methanol for 5 minutes, and nonspecific antibody binding was blocked by incubation with 1% diluted normal horse serum for 30 minutes. Sections were then incubated at 4°C overnight with the following antibodies: rabbit PGP9.5 polyclonal antibody (dilution of 1:200, Nonus Biogenesis.), rabbit NMDAR2B polyclonal antibody (dilution of 1:100, Millipore), or mouse Cyclin A monoclonal antibody (6E6, dilution of 1:50, Leica Biosystems Newcastle. Ltd). Immune complexes were detected with the Vectastain Elite ABC kit (Vector Laboratories, Inc, Burlingame, CA) according to the manufacturer’s instructions. These immune complexes were detected using the 3,3’-diaminobenzidine substrate (Vector) as a chromogen (PGP9.5 1.5 minutes, NMDAR2B 6 minutes, CCNA1 10 minutes). Sections were counterstained with hematoxylin.

### Epigenetic treatment with 5-Aza-dC and TSA, and chemotherapeutic treatment with CDDP

Cells were split and seeded at a low density (1x10^6^/T-75 flask) 24 hours before treatment. Cells were then treated every 24 hours for 4 days with either 1 or 5 μM 5-aza-2’-deoxycytidine (5-Aza-dC; Sigma-Aldrich, St Louis, MO) dissolved in 50% acetic acid or were mock treated with PBS including the same amount of acetic acid. As indicated, 100 nM of trichostatinA (TSA; Sigma-Aldrich) and/or 12.5 μM of Cis-diaminedichloroplatinum (CDDP) (Nichi-Iko Pharmaceutical Co., Ltd., Japan) was added for the final 24 hours.

### Western blotting analysis

Total protein was extracted from cell lines that were epigenetically treated with/without chemotherapeutic treatment, and was subjected to Western blotting analysis using the following antibodies: mouse anti-p53 monoclonal antibody (1C12, dilution of 1:1000, Cell Signaling Technology, Inc.) or mouse anti-β-actin IgG_2a_ monoclonal antibody (dilution of 1:10000, Sigma-Aldrich).

### p53 reporter assay

Cells (2x 10^4^ cells/96 well plate) that were epigenetically treated with/without chemotherapeutic treatment were transfected with a *p53* reporter vector (QIAGEN) using Signal™ Pathway Reporter Kits (QIAGEN) and the Lipofectamine 2000 reagent (Invitrogen). After 24 hours of incubation, the reporter activity was measured using the Dual-luciferase Reporter Assay System (Promega). Transfections were performed in triplicate and analyzed using SoftMax Pro software (Molecular Devices, Sunnyvale, CA).

### Apoptosis assay

Treated cells (1x 10^5^ cells/sample) were stained with Annexin V and 7-AAD (Guava Nexin reagent, Guava Technologies, Hayward, CA) for discrimination of early and late apoptotic cells, respectively. The experiment was carried out using the Guava PCA System, performed in triplicate and analyzed using CytoSoft 2.1.5 software (Guava Technologies).

### Statistical analysis

Fisher’s exact test or the Mann-Whitney U test was used for categorical variables, and Student’s *t*-test was used for continuous variables. Data are expressed as means± standard deviation (SD). The Kaplan-Meier method was used to estimate cumulative survival rates, and differences in survival rates were assessed using the log-rank test. Relapse free survival (RFS) and overall survival (OS) were measured from the date of operation to the date of recurrence and death, or the last follow up. With regard to RFS or OS, patients who survived for more than 60 months (5-years) were analyzed as survivors. P<0.05 was considered to indicate statistical significance. All statistical analyses were conducted with SAS software packages (SAS Institute, Cary, NC).

## Results

### *p53* gene status and methylation profiles of *PGP9*.*5*, *NMDAR2B*, *CCNA1*, and *DAPK* in pStage II-III gastric cancer

**Fig 2:**
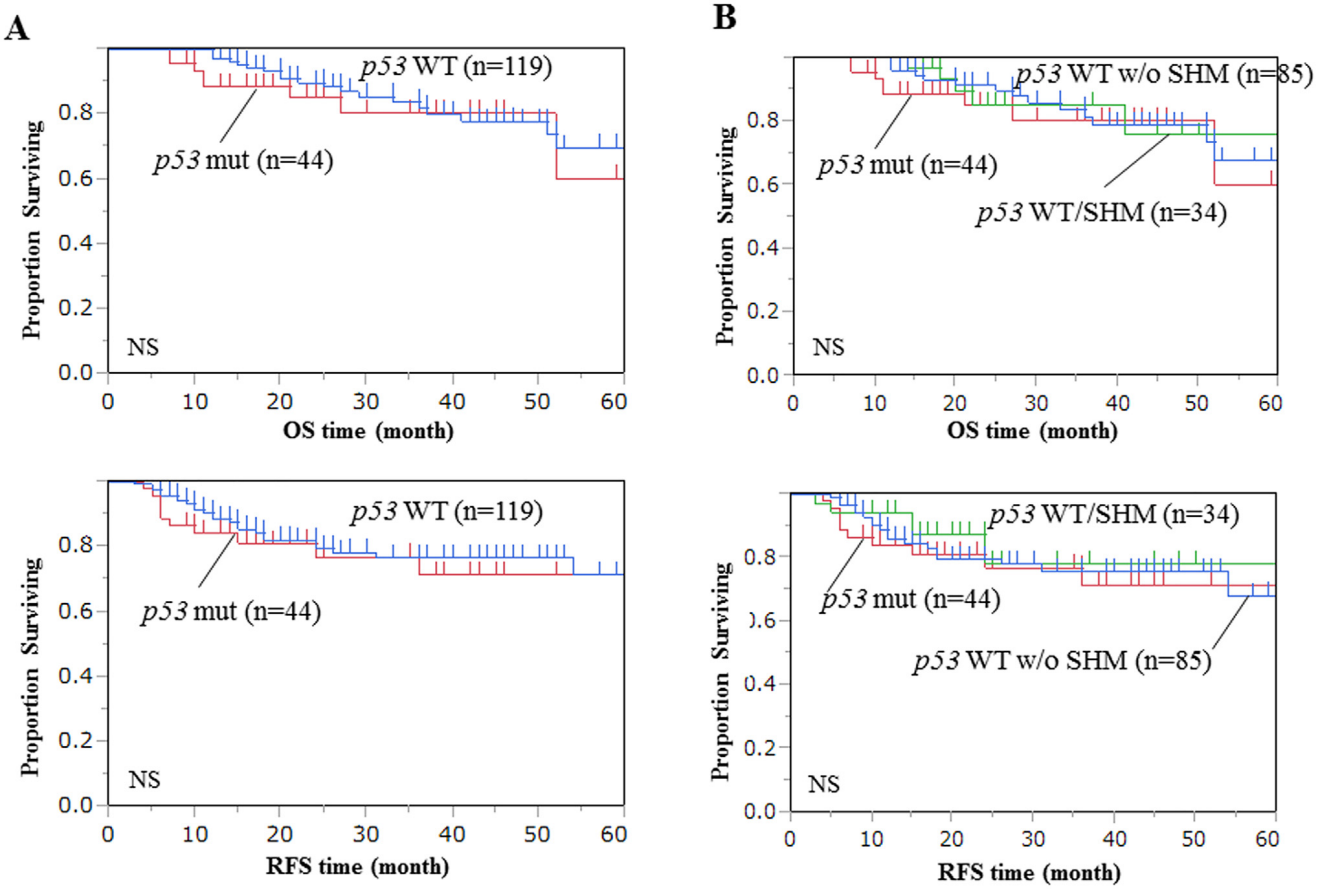
Kaplan Meier analysis of overall survival (OS) and relapse free survival (RFS) of primary gastric cancer patients with pathological stage II/III (pStage II/III) who underwent curative resection and postoperative adjuvant chemotherapy. (A) According to *p53* gene mutation status and (B) according to the *p53* aberration group.

**Fig 3:**
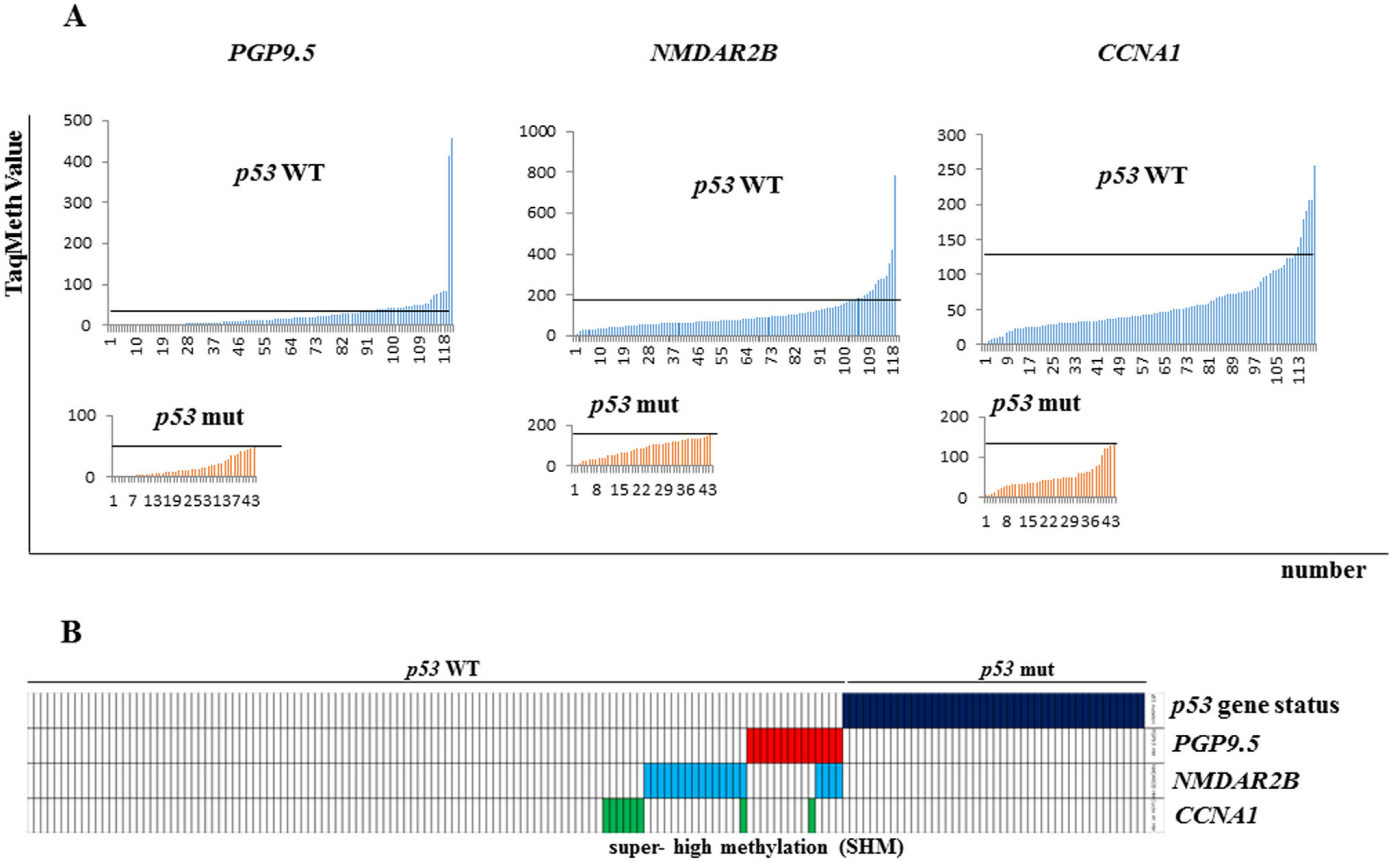
TaqMeth value of *PGP9*.*5*, *NMDAR2B*, and *CCNA1* in pStage II/III gastric cancer. (A) Methylation of the indicated genes was analyzed using Q-MSP and TAqMeth values in tumors with *p53* wild type were compared with those with *p53* mutation. The threshold value for determination that a gene was methylated was determined as the maximum TaqMeth value of *p53* wild type. (B) Tumors with *p53* wild type or *p53* mutation were compared in terms of the presence of super-high methylation of the indicated genes. Super-high methylation was defined that at least one gene showed higher TaqMeth value than each threshold values among three genes.

*p53* mutations were identified in 44 of 163 primary gastric cancer patients (27%) by SSPC analysis. *p53* gene mutation did not have prognostic relevance (Fig 2A). We then analyzed the methylation profiles of *PGP9*.*5*, *NMDAR2B*, *CCNA1*, and *DAPK* in these tissues using Q-MSP. The TaqMeth value of all genes was higher in primary tumors of gastric cancer with wild type *p53* than in those with mutant *p53* (gene methylation order: *PGP9*.*5*>*NMDAR2B*>*CCNA1*>*DAPK*) (Fig 3A and S1 and S2 Figs). Methylation of the promoter DNA was significantly higher for *CCNA1* (p<0.0001) and *PGP9*.*5* (p = 0.03), and marginally significantly higher for *NMDAR2B* (p = 0.10) and *DAPK* (p = 0.05) in primary gastric cancer compared to the corresponding normal mucosa. Importantly, a super-high methylation level of *PGP9*.*5*, *NMDAR2B*, and *CCNA1* was exclusively found in primary tumors with no *p53* mutation, the cut-off TaqMeth values were 50, 163, and 133, respectively (Fig 3A). Such super-high methylation of *PGP9*.*5*, *NMDAR2B* and *CCNA1*was found in 14, 19, and 8 samples respectively, and some of these samples were overlapping (Fig 3B). *DAPK* did not display this trend, because the *DAPK* methylation level was fairly high in the corresponding normal mucosa tissues of a considerable portion of the cases (S2 Fig).

### Immunohistochemical analysis of PGP9.5, NMDAR2N, and CCNA1 protein expression in primary gastric cancer

**Fig 4:**
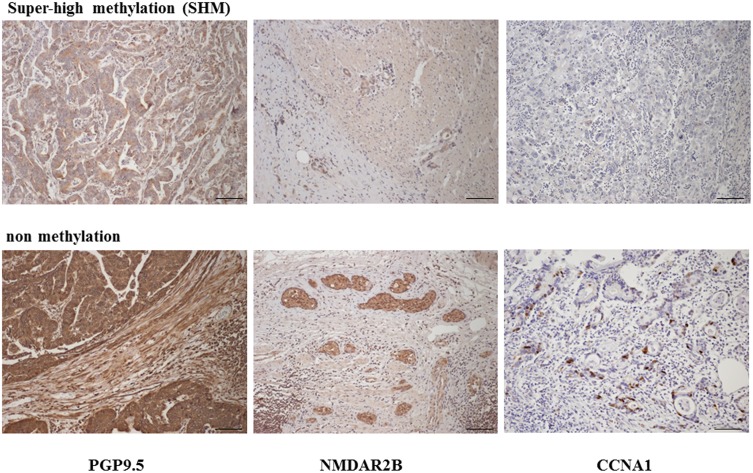
Immunohistochemical expression of PGP9.5, NMDAR2B, and CCNA1 in gastric cancer according to their methylation status. Immunohistochemical staining of PGP9.5, NMADR2B, and CCNA1 in primary tumor with or without hypermethylation of the promoter region of the corresponding gene (original magnification, X100, scale bars, 100 μm).

Immunohistochemical staining for PGP9.5, NAMDR2B and CCNA1 protein expression was then carried out both in cases with super-high methylation and those with low methylation of these genes. A strong reduction in the expression of PGP9.5, NMDAR2B, and CCNA1 was observed in primary gastric cancer tissues when the promoter DNA methylation was super-high (Fig 3). On the other hand, no decrease in the protein expression of PGP9.5, NMDAR2B, or CCNA1 was found when the promoter DNA methylation was low (Fig 4).

### Clinicopathological analysis in primary gastric cancer with pathological stage II/III gastric cancer with standard treatment

Clinicopathological features and prognosis (5-year RFS and OS) were then analyzed in a univariable manner in gastric cancer with pathological stage II/III gastric cancer with standard treatment (surgery plus postoperative S-1 administration) (Table 1). We classified the gastric cancer patients into 3 categories based on the *p53* mutation status as well as the DNA methylation status of *PGP9*.*5*, *NMDAR2B* and *CCNA1*, which we designated as genomic and epigenetic categories, respectively (GEC). These three categories were *p53* mutant, *p53* wild type with super-high methylation (SHM) of the above 3 *p53* pathway genes (*p53* WT/SHM), and *p53* wild type without (w/o) SHM (*p53* WT w/o SHM).

Interestingly, patient groups classified based on GECs were significantly correlated with gender (p = 0.0003), age (p = 0.08), Lauren’s histology (p = 0.005) and infiltration pattern (p = 0.001), but not with prognostic factors such as staging factors. Patient groups classified based on GECs also showed that *p53* mutant and *p53* WT/SHM groups were similar in terms of the patient number for each clinical characteristic but differed in this respect when compared to the *p53* WT w/o SHM group. Thus, we newly designated the former groups (the *p53* mutant plus the *p53* WT/SHM groups) as the *p53* aberration group, and the latter group (*p53* WT w/o SHM) was designated as the *p53* non-aberration group.

Although prognosis was not significantly different among the groups categorized according to GEC (Fig 2B), more recurrences were found in the *p53* aberration group as compared to the *p53* non-aberration group (Table 1, no statistically significant difference). This trend was preserved in terms of the recurrence pattern of each of the lymph nodes, peritoneum, and distant organs (Table 2), suggesting that the *p53* aberration group is likely to exhibit a clinically unique phenotype even from a prognostic point of view. When only cases with recurrences were analyzed, the *p53* aberration group was again significantly associated with Lauren’s histology (Table 2, P = 0.01), however there were no recurrence patterns that were unique to *p53* aberration.

**Table 2:** Clinicopathological characters of recurrent tumors of pStage II/III gastric cancer in the *p53* aberrant group (A) and the *p53* non-aberrant group (B).

	*p53* aberration group	*p53* non-aberration group	P value
variable	n = 16	n = 18	
**Gender**			**NS**
** Male**	**15**	**13**	
** Female**	**1**	**5**	
**Age**			**NS**
** <67**	**4**	**9**	
** ** **≥** **67**	**12**	**9**	
**Lauren's histology**			**0.01**
** Diffuse type**	**5**	**14**	
** Intestinal type**	**11**	**4**	
**INF**			**NS**
** α**	**2**	**1**	
** β**	**9**	**4**	
** γ**	**5**	**13**	
**Recurrent site**			**NS**
** lymph node**	**7**	**8**	
** peritneum**	**4**	**5**	
** distant organ**	**4**	**5**	
** anastomosis**	**1**	**0**	
**Recurrent time (month)**			**NS**
** median (range)**	**8.5 (3–36)**	**11.5 (5–54)**	

NS: not significant.

aberration: *p53* mutation and *p53* WT/SHM.

non-aberration: *p53* WT w/o SHM.

**Fig 5:**
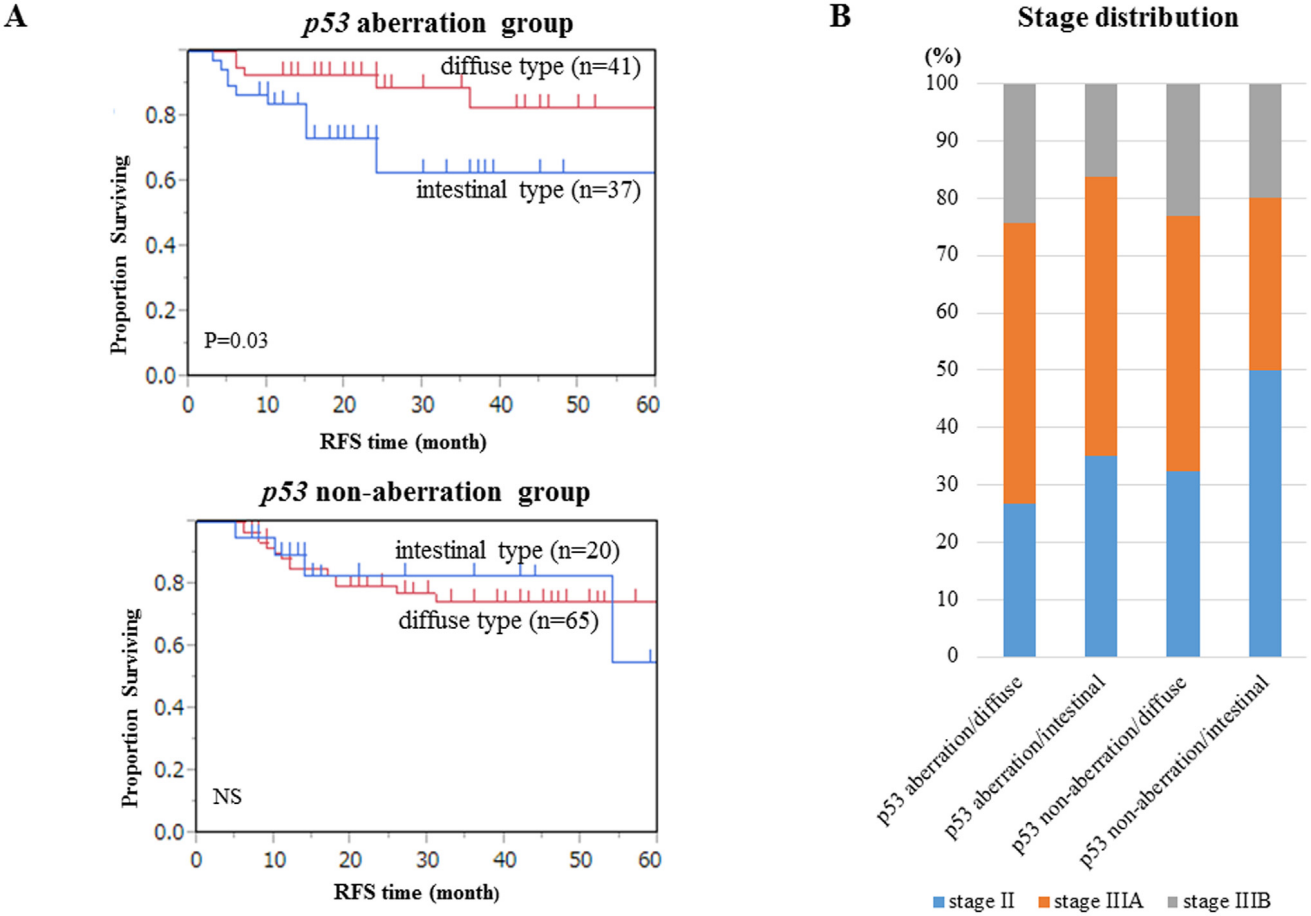
Kaplan-Meier analysis of 5 year RFS and Stage distribution in pStage II/III gastric cancer in the *p53* aberrant and *p53* non-aberrant groups. (A) Survival curves for intestinal type were compared with those of diffuse type gastric cancer with pStage II/III. (B) Cancer stage distribution according to the *p53* aberration group and histological findings.

On the other hand, the diffuse type of gastric cancer showed significantly better prognosis than the intestinal type of gastric cancer in the *p53* aberration group (Fig 5A). Since such patients tended to be distributed to a more advanced stage, these survival data suggested that S1 postoperative adjuvant chemotherapy is more effective for diffuse type gastric cancer than for intestinal type gastric cancer in cases with *p53* pathway aberration (Fig 5B).

Finally, as reference data (Table 1), univariable prognostic analysis for RFS and OS of all patients identified clinically significant potential prognostic factors representing poor survival such as gender (P = 0.03, P = 0.1), age ≥67 years (P = 0.009, P = 0.001), upper tumor location (P = 0.06, P = 0.1), the 14^th^ JGCA/7^th^ UICC pT (P = 0.08, P = 0.4), the 14^th^ JGCA pN (P = 0.001, P = 0.06), and the 14^th^ JGCA/7^th^ UICC stage (P<0.0001, P = 0.03).

### Epigenetic treatment induced p53 protein expression, concordant with p53 transcriptional activity

**Fig 6:**
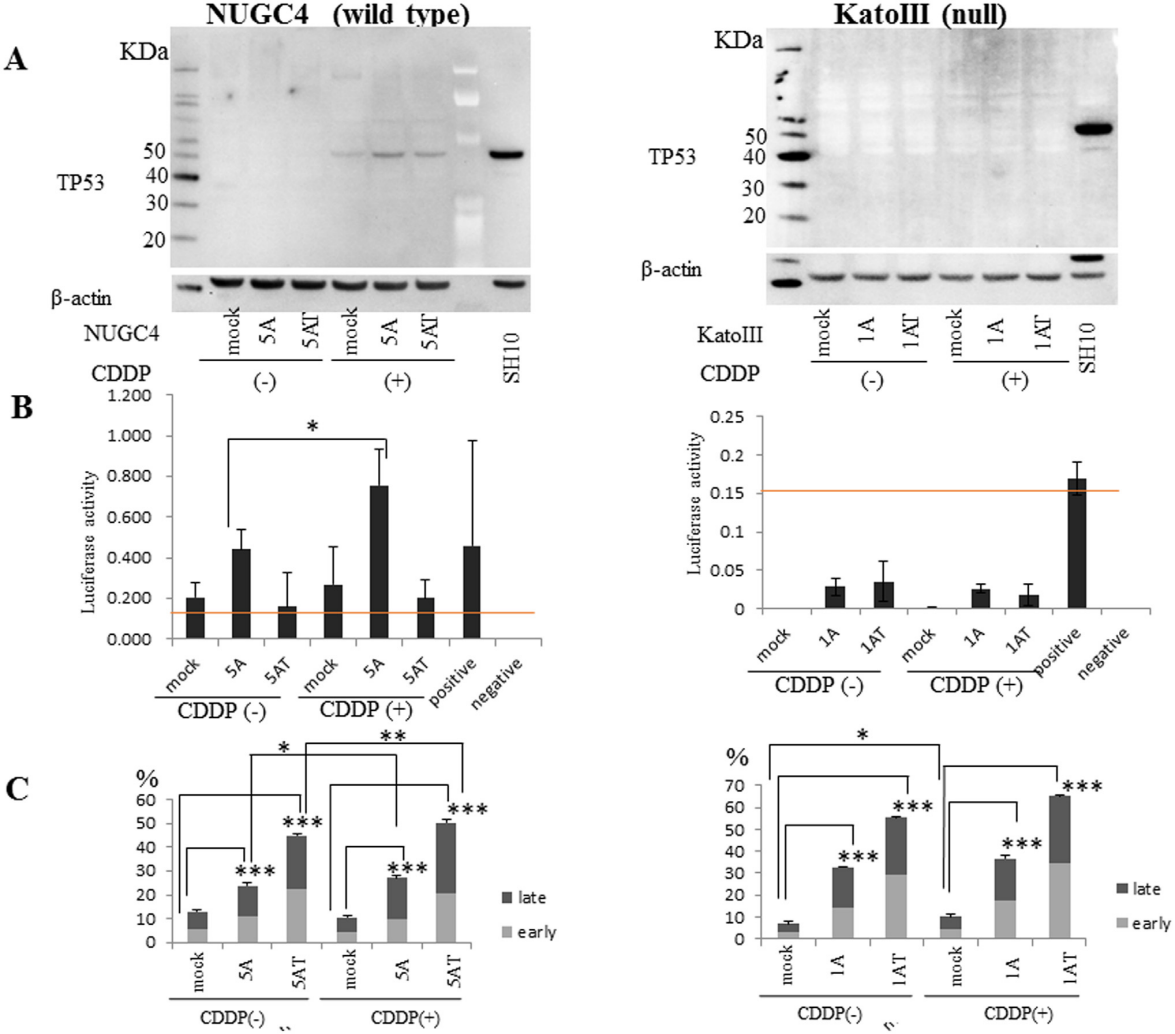
Epigenetic and/or chemotherapeutic treatments of the NUGC4 (wild type *p53*) and KATOIII (*p53* null) gastric cancer cell lines. NUGC4 and KATOIII cells were treated with the demethylating agent, 5-aza-2’-deoxycytidine (5-aza-dC) in the presence or absence of the histone deacetylase (HDAC) inhibitor, trichostatin A (TSA), and in the presence or absence of the chemotherapeutic reagent, CDDP. 1A and 5A, 1 and 5 μM 5-aza-dC; T, TSA. Subsequently the cells were analyzed for: (A) p53 protein expression by Western Blotting. (B) A dual reporter assay to confirm *p53* transcription activity. The dashed line indicates the optimal cut-off value (0.15) for determination of p53 activity. (C) Cell apoptosis was assayed using a Nexin Assay. A representative image is shown. Data are shown as the percentage of early and late apoptotic cells. *P<0.05, **<0.001, ***<0.0001.

Epigenetic treatment of NUGC4 gastric cancer cell lines (wild type *p53*) with 5 μM 5-aza-2’-deoxycytidine or 5 μM 5-aza-2’-deoxycytidine plus trichostatin A robustly induced apoptosis in the absence of the chemotherapeutic agent CDDP. Additional treatment with CDDP further significantly augmented these apoptotic effects (Fig 6C). Interestingly, CDDP in combination with epigenetic treatments robustly increased the p53 protein level, which partially, but not totally, reflected *p53* transcriptional activity of a luciferase reporter gene (Fig 6A and 6B). These findings suggested that *p53* transcription activity can be reactivated by epigenetic treatments, however it also suggests that apoptosis is only partially induced through the *p53* pathway.

Moreover, epigenetic treatment of KATOIII gastric cancer cell lines (*p53* null), with 1 μM 5-aza-2’-deoxycytidine or 1 μM 5-aza-2’-deoxycytidine plus trichostatin A robustly induced apoptosis in the absence of CDDP, but additional CDDP treatment did not further significantly augment the apoptotic effects (Fig 6C). CDDP in combination with epigenetic treatments did not increase the p53 protein level, which was reflected by the lack of *p53* transcriptional activity of a luciferase reporter gene (Fig 6A and 6B). These findings suggested that *p53* transcription activity is not required for apoptosis by epigenetic treatments in KATOIII cells (*p53* null cells).

## Discussion

This current study is the first report that SHM of tumor suppressor genes in the *p53* pathway are found exclusively in pathological stage II/III gastric cancer with wild type *p53* (Fig 3). We selected pStage II/III gastric cancer patients with standard treatments for *p53* pathway analysis, because multidisciplinary treatments in such patients have the best potential for attaining prognostic improvement with complete curability [24, 25]. This consideration should be the first priority when considering further development of novel therapeutic strategies.

Almost complete methylation of promoter DNA CpG islands is required for complete gene silencing, and even small proportions of unmethylated alleles can robustly induce gene expression [1–5]. In the primary tumor tissues, SHM must represent almost complete methylation in cancer cells, and SHM in the primary tumor tissues was indeed associated with reduced protein expression of such genes (Fig 4). This result suggested that SHM of genes in the *p53* pathway could be functionally equivalent to *p53* functional aberration that occurs as a result of *p53* mutation, since these 3 molecules function in the same *p53* pathway.

In gastric cancer, the *p53* aberration group was significantly associated with unique clinicopathological phenotypes such as Lauren’s intestinal histology, male gender, old age, and less infiltrative growth (Table 1). Our current observations may be related to previous reports that demonstrated that *p53* mutation is correlated with early stage of intestinal type gastric cancer, or with late stage of diffuse type gastric cancer [26, 27], or elderly age [28], however, our clinicopathological analysis mainly included middle stage gastric cancer. Interestingly, pathway aberration is unique and sustained even in recurrent patients (Table 2).

Positive prognostic relevance of *p53* mutation in gastric cancer is controversial with both supporters [29] and dissenters [30] of this possibility. At first glance our data would appear to support the latter group, however, it should be taken into account that our survival data is likely to have been modified by post-operative adjuvant chemotherapy (standard therapy in Japan). Whereas, in contrast to the present study, the diffuse type of gastric cancer showed poorer prognosis than the intestinal type of gastric cancer in our hospital decades ago [15, 31], the latest updated survival data support the opposite results (i.e., the intestinal type of gastric cancer shows poorer prognosis than the diffuse type of gastric cancer), which is putatively due to S-1 postoperative adjuvant effects [32]. Post-operative adjuvant S-1 chemotherapy has been proposed to be significantly more effective for peritoneal disease than for distant organ metastasis [24], and it is likely to be more effective for diffuse type gastric cancer than for intestinal type gastric cancer [32]. For these reasons, the latest survival analysis indicates that the prognosis of the diffuse type of gastric cancer is improved over that of the intestinal type of gastric cancer.

There was no statistical difference in the recurrence rate or in the unique recurrence sites between the *p53* aberration group and the *p53* non-aberration group, however more recurrences were found in each of the recurrence sites of the *p53* non-aberration group compared to the *p53* aberration group. These findings might suggest that the *p53* non-aberration group has a more aggressive phenotype than the *p53* aberration group as a whole. In our study, the diffuse type of gastric cancer showed significantly better prognosis than the intestinal type of gastric cancer in the *p53* aberration group. Since such patients tended to be distributed to a more advanced stage, these survival data suggested that S1 postoperative adjuvant chemotherapy is more effective for diffuse type gastric cancer than for intestinal type gastric cancer in cases with *p53* pathway aberration. These findings were consistent with previous reports that mutant *p53* and/or immunostaining of p53 protein could be predictive biomarkers for positive effects of high-dose neoadjuvant chemotherapy in gastric cancer [30].

We finally assessed epigenetic treatment effects in combination with a chemotherapeutic agent in order to compare the importance of the *p53* pathway to the epigenetic pathway in gastric cancer cells treated with CDDP. Unexpectedly, it appeared that the *p53* pathway was unlikely to play a very critical role in apoptosis induction by CDDP (Fig 6). It was recently demonstrated that epigenetic carcinogenesis is indeed possible. In this system, systemic iPS induced by reprogramming factors results in systemic cancer occurrence with dynamic epigenetic changes, while systemic reversion of these reprogramming factors reverts cancer cells into normal cells [33]. Gastric cancer is likely to harbor fewer mutations of driver genes [34] than colorectal cancer [35], and, similarly, mutations other than the *p53* gene are rare in breast cancer [35], suggesting that epigenetic carcinogenesis is a possible major etiology through chronic infection of Helicobacter Pylori [36–38]. We have recently demonstrated the hyper-methylation of *Reprimo* [39], which again is a gene in the *p53* pathway but which could not be included in this study, and of *HOPX* [16] and *CDO1* [40] that are both involved in apoptosis, but putatively not through the *p53* pathway. Based on our current functional experiments, the *p53* pathway contribution to epigenetic treatment effects is likely to be much less than that of other pathways (Fig 5). We used other cell lines (2 gastric cancer cell lines and 1 hepatocellular cancer cell line) to accurately determine the contribution of the *p53* pathway to apoptosis induced by CDDP in combination with epigenetic treatments. The additional experiments performed are shown in S3 Fig. AZ521 and HepG2 cell lines are *p53* wild type, and the SH10 cell line is *p53* mutant. *p53* activity was detected in the *p53* wild type cells, however, its activity was dependent on each cell line. Thus, *p53* activity was detected in NUGC4 cells with CDDP treatment and in AZ521 and HepG2 cells without CDDP treatment. Strong apoptosis as assessed by an apoptosis assay (Caspase 3 or Nexin Assay) did not necessarily reflect the degree of *p53* transcriptional activation. In *p53* mutant (SH10) or *p53* null cells, *p53* transcriptional activity was not induced at all, however apoptosis was found to be similar to that in *p53* wild type cells. Therefore, in gastric cancer, apoptotic pathways other than the *p53* pathway may be more relevant for induction of apoptosis than the *p53* pathway.

In conclusion, *p53* aberration and non-aberration patient groups exist in gastric cancer, the *p53* pathways could be aberrant as a result of epigenetic modification of genes in the pathways as well as due to genomic mutation. The *p53* aberration group may exhibit unique clinical features as compared with the *p53* non-aberration group. Epigenetic control of the *p53* pathway genes play a minor role in apoptosis induction by a chemotherapeutic agent, and apoptosis in gastric cancer that is induced by epigenetic reversion may be explained largely by mechanism other than the *p53* pathway. Thus, the *p53* pathway is unlikely to play a pivotal role in apoptosis induced by CDDP in combination with epigenetic treatments. However, the *p53* pathway has been reported to play important roles in natural gastric carcinogenesis [41–44]. Thus, our discovery that *p53* pathway gene methylation was exclusively found in gastric cancer with a *p53* wild type status may be an important observation, and such alterations in the tumor tissues may cause tumor progression. It is for these reasons that *p53* pathway aberration may have predictive value of chemotherapeutic effects in gastric cancer.

## Supporting Information

S1 Fig Specificity of the methylation of *PGP9*.*5*, *NMDAR2B*, and *CCNA1* in gastric cancer. (A) The TaqMeth values of each gene in gastric cancer and in the corresponding normal mucosa are shown. (B) The TaqMeth values of each gene classified according to the *p53* gene status of the tumor are shown. Data are expressed as means ± SD.
(TIF)

S2 Fig TaqMeth value of *DAPK* in gastric cancer. (A) TaqMeth value of *DAPK* in gastric cancer and the corresponding normal mucosa. (B) TaqMeth value of *DAPK* classified according to *p53* gene status. (C) The TaqMeth value was slightly higher in primary gastric cancer tumors with *p53* wild type than in those with *p53* mutation.
(TIF)

S3 Fig Epigenetic treatments of the gastric cancer cell line AZ521, the Hepatocellular cancer cell line HepG2 (wild type *p53*), and the gastric cancer cell line SH10 (*p53* mutant). After treatment of AZ521, HepG2 and SH10 cells with 5-aza-dC in the presence or absence of TSA, and in the presence or absence of CDDP. (A) p53 protein expression was assayed by Western Blotting. (B) A dual reporter assay was performed to confirm *p53* transcription activity. The dashed line indicates the optimal cut-off value (0.15) for determination of p53 activity. (C) Cell apoptosis was assayed and representative images are shown. Apoptosis of AZ521 and HepG2 cell lines were measured using a Caspase 3 Assay. Caspase 3 activity was measured with Caspase Glo3/7 Assay (Promega) according to the manufacturer’s recommendations. Apoptosis of the SH10 cell line was measured using a Nexin Assay. *P<0.05, **<0.001, ***<0.0001.
(TIF)

S1 Table Q-MSP production and sequence of primers and fluorescent probe. (XLSX)

S2 Table Clinical raw datas which were coded. (XLSX)
