# Supplementary figures and images for: Exclusive Association of p53 Mutation with Super-High Methylation of Tumor Suppressor Genes in the p53 Pathway in a Unique Gastric Cancer Phenotype
Source: PLoS One. 2015 Oct 8;10(10):e0139902. doi: 10.1371/journal.pone.0139902 (PMC4598091; doi:10.1371/journal.pone.0139902)

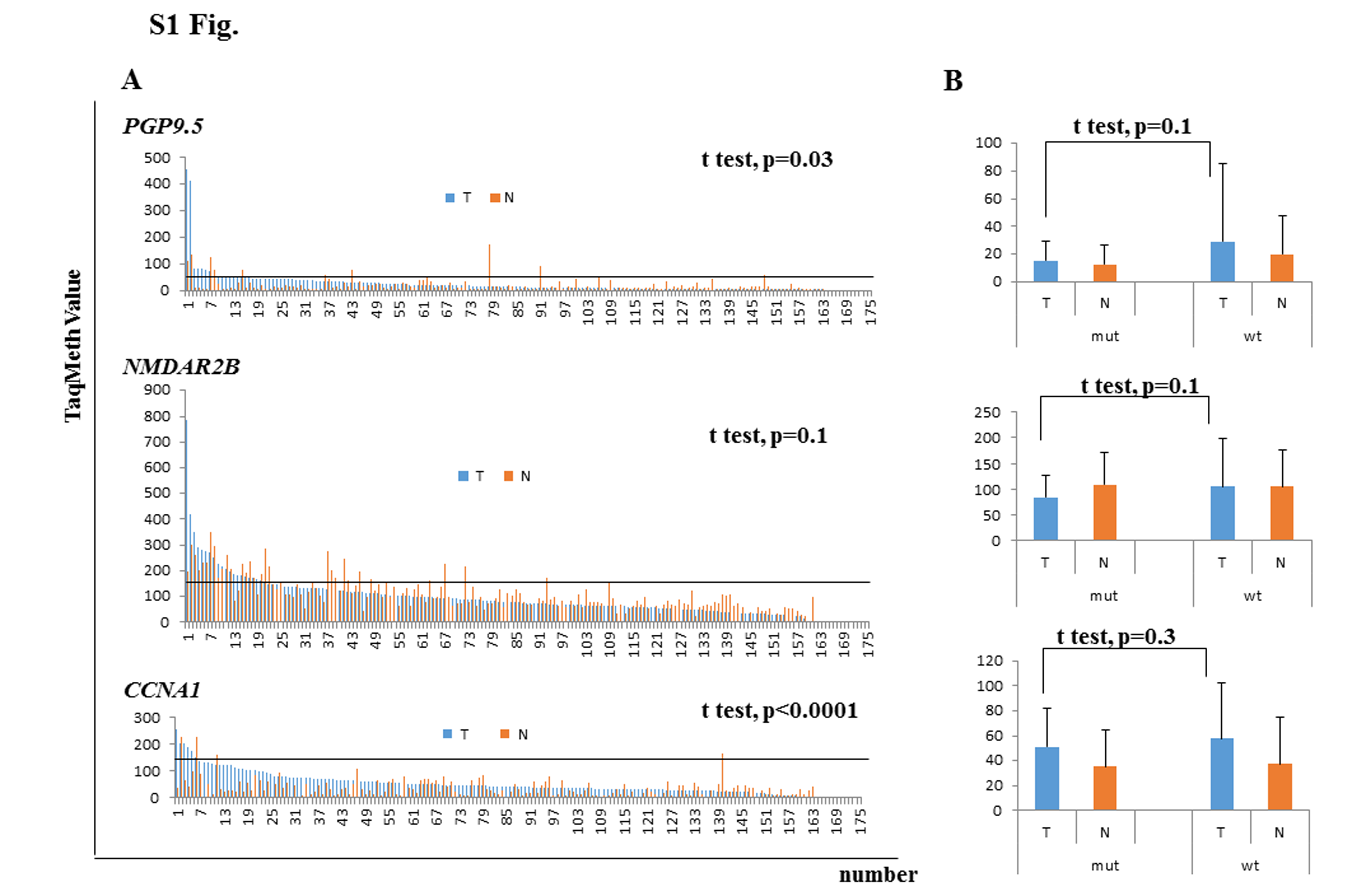

Supplement: S1 Fig — (A) The TaqMeth values of each gene in gastric cancer and in the corresponding normal mucosa are shown. (B) The TaqMeth values of each gene classified according to the p53 gene status of the tumor are shown. Data are expressed as means ± SD. (TIF) [file pone.0139902.s001.tif]

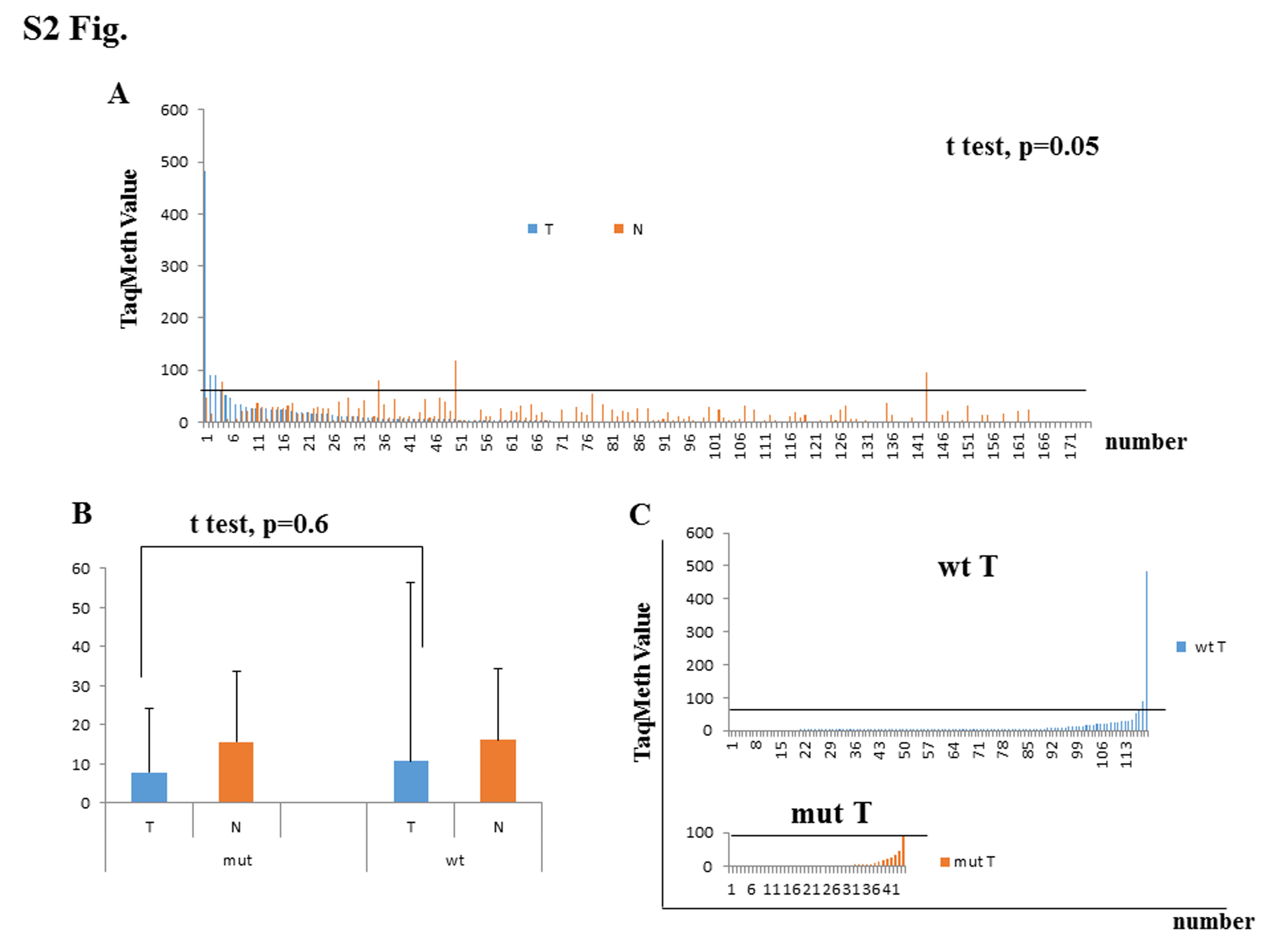

Supplement: S2 Fig — (A) TaqMeth value of DAPK in gastric cancer and the corresponding normal mucosa. (B) TaqMeth value of DAPK classified according to p53 gene status. (C) The TaqMeth value was slightly higher in primary gastric cancer tumors with p53 wild type than in those with p53 mutation. (TIF) [file pone.0139902.s002.tif]

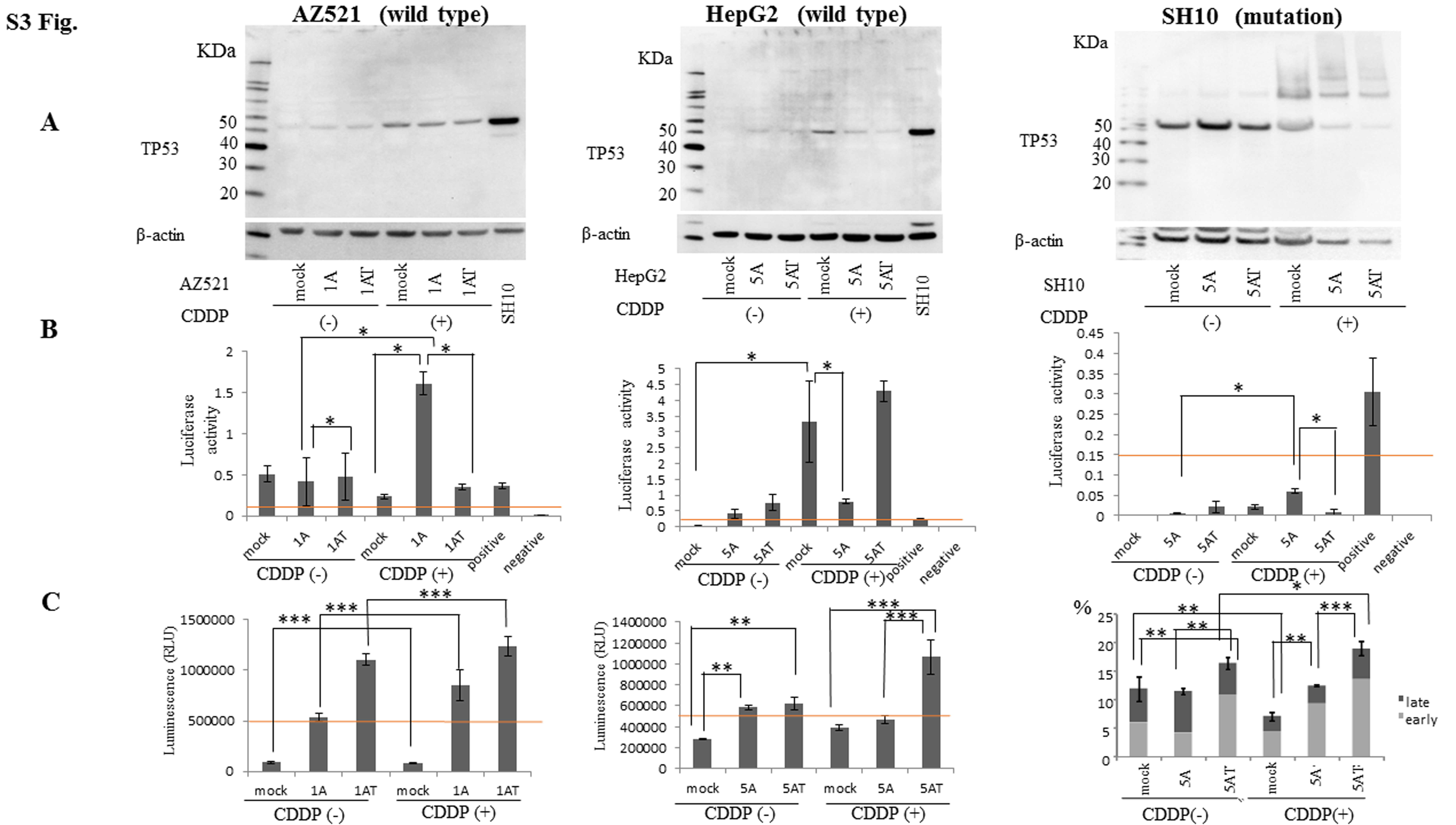

Supplement: S3 Fig — After treatment of AZ521, HepG2 and SH10 cells with 5-aza-dC in the presence or absence of TSA, and in the presence or absence of CDDP. (A) p53 protein expression was assayed by Western Blotting. (B) A dual reporter assay was performed to confirm p53 transcription activity. The dashed line indicates the optimal cut-off value (0.15) for determination of p53 activity. (C) Cell apoptosis was assayed and representative images are shown. Apoptosis of AZ521 and HepG2 cell lines were measured using a Caspase 3 Assay. Caspase 3 activity was measured with Caspase Glo3/7 Assay (Promega) according to the manufacturer’s recommendations. Apoptosis of the SH10 cell line was measured using a Nexin Assay. *P<0.05, **<0.001, ***<0.0001. (TIF) [file pone.0139902.s003.tif]
